# Deep learning-assisted ultrasonic diagnosis of cervical lymph node metastasis of thyroid cancer: a retrospective study of 3059 patients

**DOI:** 10.3389/fonc.2024.1204987

**Published:** 2024-02-08

**Authors:** Hai Na Zhao, Hao Yin, Jing Yan Liu, Lin Lin Song, Yu Lan Peng, Bu Yun Ma

**Affiliations:** ^1^ Department of Ultrasonography, West China hospital of Sichuan University, Chengdu, Sichuan, China; ^2^ Computer science of Sichuan University, Chengdu, Sichuan, China

**Keywords:** deep learning-assisted ultrasonic diagnosis, cervical lymph node metastasis, thyroid cancer, retrospective, fine needle aspiration sensitivity, specificity, accuracy, PPV

## Abstract

**Objective:**

This study aimed to develop a deep learning system to identify and differentiate the metastatic cervical lymph nodes (CLNs) of thyroid cancer.

**Methods:**

From January 2014 to December 2020, 3059 consecutive patients with suspected with metastatic CLNs of thyroid cancer were retrospectively enrolled in this study. All CLNs were confirmed by fine needle aspiration. The patients were randomly divided into the training (1228 benign and 1284 metastatic CLNs) and test (307 benign and 240 metastatic CLNs) groups. Grayscale ultrasonic images were used to develop and test the performance of the Y-Net deep learning model. We used the Y-Net network model to segment and differentiate the lymph nodes. The Dice coefficient was used to evaluate the segmentation efficiency. Sensitivity, specificity, accuracy, positive predictive value (PPV), and negative predictive value (NPV) were used to evaluate the classification efficiency.

**Results:**

In the test set, the median Dice coefficient was 0.832. The sensitivity, specificity, accuracy, PPV, and NPV were 57.25%, 87.08%, 72.03%, 81.87%, and 66.67%, respectively. We also used the Y-Net classified branch to evaluate the classification efficiency of the LNs ultrasonic images. The classification branch model had sensitivity, specificity, accuracy, PPV, and NPV of 84.78%, 80.23%, 82.45%, 79.35%, and 85.61%, respectively. For the original ultrasonic reports, the sensitivity, specificity, accuracy, PPV, and NPV were 95.14%, 34.3%, 64.66%, 59.02%, 87.71%, respectively. The Y-Net model yielded better accuracy than the original ultrasonic reports.

**Conclusion:**

The Y-Net model can be useful in assisting sonographers to improve the accuracy of the classification of ultrasound images of metastatic CLNs.

## 1 Introduction

Primary thyroid cancer (TC) is the most common malignant endocrine tumor worldwide, and its incidence has steadily increased over the past two decades (1, 2). TC has often metastasized to the cervical lymph nodes (CLN) at the time of diagnosis, with more than half of the patients with TC having lymph node (LN) metastasis at the initial diagnosis (3, 4). Accurate evaluation of CLN metastasis is important for TC patients, not only for surgical approach selection, but also in relation to long-term clinical outcomes. Ultrasound (US) is one of the most important imaging methods for evaluating TC nodule and CLN metastasis. However, due to the complex structure of the neck and the diverse ultrasound manifestations of LNs, the US examination depends more on the operator experience and their subjective impression (5–7). Therefore, a new strategy is required to overcome operator dependence.

Artificial intelligence (AI)-assisted diagnosis can reduce US operator dependence and in recent years, it has become popular in ultrasonic diagnosis. As a subdomain of AI, a convolutional neural network (CNN) can extract image features and is widely used for ultrasonic image classification (8, 9). Previous research reports have mostly focused on predicting the risk of CLN metastasis based on the US characteristics of thyroid nodules (10, 11), and there are few deep learning studies in differentiating benign and malignant CLN based on CLN US images in TC cases. Therefore, in this study, we used a CNN named Y-Net to automatically segment and classify CLN US images, which can assist radiologists in more accurate analyses and decisions regarding fine needle aspiration (FNA) during US examinations.

## 2 Materials and methods

### 2.1 Ethical approval

This study was approved by the Ethics Committee of West China Hospital, Sichuan University (No. 1341). Written informed consent was not required owing to the retrospective nature of this study. All datasets were fully anonymized.

### 2.2 Patients and datasets

Patients who visited the West China hospital of Sichuan university for CLN FNA from January 2014 to December 2020 were enrolled in this study. Both preoperative and postoperative CLNs were included. During FNA, the puncture route avoided the thyroid parenchyma. The washout thyroglobulin (Tg) levels were considered positive only if they were significantly higher than the serum Tg levels. All CLNs were confirmed using FNA and/or washout Tg analyses.

The inclusion criteria were as follows:

1. Patients suspected with TC metastasis.
2. Patients with high-quality B-mode and color Doppler images using a high-frequency linear probe during the examination.
3. Patients with pathologically confirmed CLNs.

The exclusion criteria were as follows:

1. Patients with LNs not located in the neck.
2. Patients with indistinct ultrasonic images.
3. Patients with indeterminate final pathological results.

All images were collected by a sonographer with eight years experience. US imaging was performed using the following five different US machines for data acquisition: Mindray Resona 7T (Mindray Medical International, Shenzhen, China), Siemens Acuson Oxanal (Siemens Medical Systems, Munich, Germany), Philips IU22 (Philips Healthcare, Bothell, USA), Hitachi-HI Vision Preirus (Hitachi Aloka Medical, Ltd., Tokyo, Japan), and Supersonic Aixplorer (SuperSonic Imagine, Provence, France). B-mode US images of the long-axis sections of the LNs were selected for deep learning analysis. One image was captured per LN. If a patient underwent repeated examination before surgery, the most typical image was collected. To obtain high-quality images, sonographers adjusted the machine settings, such as depth, focus location, gain, and magnification, during the examination.

### 2.3 Diagnostic efficacy evaluation of original ultrasound reports

Text data from the original US reports were derived. Because the US reports were described using natural language, we first established an US diagnosis dictionary according to the principle of similar words and synonyms. Natural words such as LN enlargement, abnormal LN, and metastasis were classified as malignant, whereas natural keywords such as normal LN and reactive hyperplastic LN were classified as benign. The results are summarized in **Table 1**. Therefore, all LNs were ultrasonically divided into benign and malignant. Using pathological results and/or washout Tg analysis as the gold standard, we evaluated the diagnostic efficacy of LNs using the original US report.

**Table 1:** Diagnosis dictionary of the original ultrasonic reports.

Dictionary	Definition	Key words in the ultrasonic reports
Final diagnosis	Malignancy	Lymphadenopathy / abnormal lymph node / metastasis / CA / Ca / suspicious calcification/ suspicious necrosis
	Benignancy	Normal lymph node / reactive hyperplasia / normal structure

### 2.4 Ultrasonic image manual annotation

B-mode US images were acquired. Because all the included patients underwent US-guided percutaneous FNA and images were obtained during each procedure, we could easily determine the target CLN according to the position of the needle tip.

### 2.5 Deep learning model

**Figure 1:**
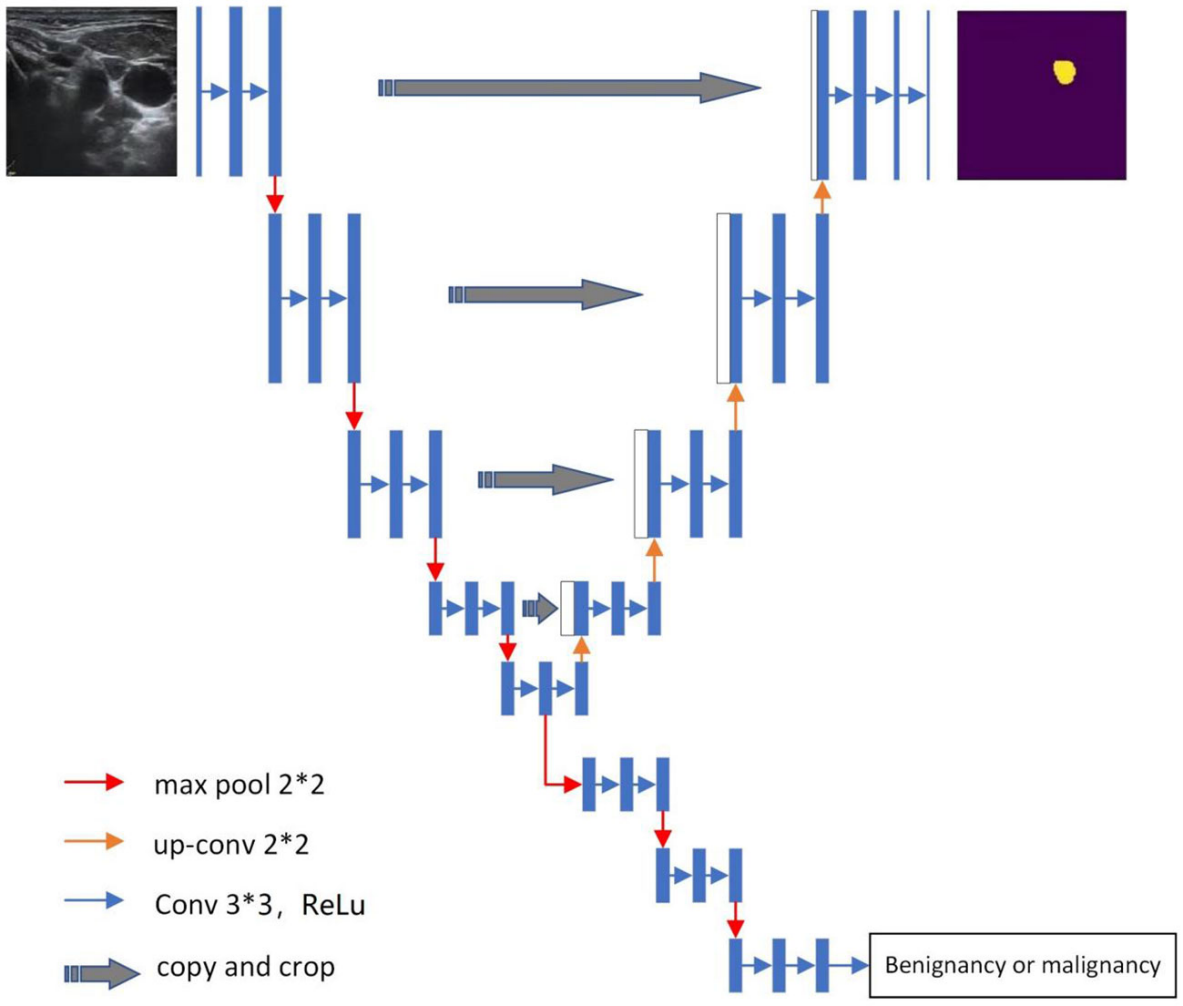
The Y-Net architecture. * means "superposition".

In this study, the Y-Net deep learning model was used (**Figure 1**). This model extends the U-Net neural network model by adding a parallel branch that can realize automatic segmentation and classification. Different from the U-Net model, we used residual convolutional blocks and efficient spatial pyramid blocks for encoding and decoding (12, 13).

① The US image exported from the ultrasonic workstation was marked with machine parameters and body markers at the corner of the image, and preconditioning was performed on each image to remove irrelevant information. We used the Mouse software designed by our research team to annotate the region of interest of the image. The US images of all desensitized LNs were scaled to a unified size of 256 × 256.
② The model encoder used an efficient pyramid module to replace the 3 × 3 convolution in the residual network. The pyramid pooling module fused features at four different pyramid scales. According to the size of the US image, our pyramid pooling module was a four-level module with bin sizes of 3 × 3, 5 × 5, 7 × 7, and 9 × 9. We processed the spatial information of the multiscale input feature map to effectively establish the long-term dependency between multiscale channel attention.
③ We added pyramid spatial pooling blocks for decoding based on the success of PSPNet for segmentation (14).
④ This model follows the characteristics of the U-Net model (14) and adds a jump connection between the encoding and decoding layers. The difference is that Y-Net also adds a jump connection between the first and last encoding block at the same spatial resolution in the encoder to improve segmentation.
⑤ The model has two parallel branches of segmentation and classification that can automatically generate segmented LN US images and LN benign and malignant classification results simultaneously. The segmented image was saved as a binary image, and the classification result was a C-dimensional vector, which was generated as a 0–1 dataset.
⑥ The cutoff value of the classification efficiency with a receiver operating characteristic (ROC) curve was determined, and the classification efficiency of the model was evaluated.

### 2.6 Loss function

To train the network, we used cross-entropy as the loss function for both segmentation and classification, which is defined as follows (Equation 1):


(1) $$J(\theta )=-\frac{1}{m}[\sum_{i=1}^{m}y^{\left(i\right)}\log f_{\vartheta }(I^{\left(i\right)})+(1-y^{\left(i\right)})log(1-f_{\vartheta }(I^{\left(i\right)}))]$$

When training segmentation, m represents the predictive category, $y^{\left(i\right)}$ the category label, and i zero or one, where zero represents benignancy and one represents malignancy. ϑ represents the consent of the network and $f_{\vartheta }\left(\cdot \right)$ the mapping relationship from the input image to the predicted output.

When training classification, m represents the predictive category, $y^{\left(i\right)}$ the ROC of each US image, and i zero or one, where zero represents the image area outside the ROC and one the ROC. ϑ represents the consent of the network and $f_{\vartheta }\left(\cdot \right)$ the mapping relationship from the input image to the predicted output.

In this study, the total loss function was segmentation combined with classification loss function.

The training process of the deep learning model was as follows. First, the patients were divided into two groups, in which the images of approximately four of five patients were used as the training set and those of the other patient, used as the validation set. Second, all the cases were randomly divided as training set and test set. A 5-fold cross-validation was used to train the parameters of the model based on the training set. The test set of completely different cases was used to validate the performance of the final model.

The computer system used to train the model was Windows 10, the central processing unit was core i7-8700, the internal storage was 8 G, and the memory of the hard disk was 1 T. The batch size was set to eight, and the initial learning rate was set to 0.001.

In this study, we evaluated the efficiency of segmentation combined with classification. We also evaluated the efficiency of only classification without segmentation of the Y-Net model by deleting the image segmentation program part. The input images were normalized the same as previous analysis. The machine parameters and body markers at the corner of the US images were removed, and the input features were also scaled to a unified size of 256 × 256. Scaling and enhancement algorithms were used to normalize them.

### 2.7 Statistical analyses

Segmentation efficiency: We assessed the automatic segmentation accuracy of the Y-Net network model using the manual segmentation result as a standard. The area similarity coefficient of the Dice similarity coefficient (DSC) was used to assess the automatic segmentation. The DSC was calculated as the ratio of the overlap area between automatic and manual segmentations to the union of automatic and manual segmentations, which is defined as follows (Equation 2):


(2) $$DSC=\frac{2\left|A_{pred}\cap A_{gt}\right|}{\left|A_{pred}\right|\cup \left|A_{gt}\right|}$$

where *A_pred_* is the area of automatic segmentation and *A_gt_* is the area of manual segmentation. The larger the DSC coefficient, the better the efficiency of the segmented model. In each case, DSC was used using Python.

Classification efficiency was evaluated in terms of sensitivity (Sen) (Equation 3), specificity (Spe) (Equation 4), accuracy (Acc) (Equation 5), positive predictive value (PPV) (Equation 6), negative predictive value (NPV) (Equation 7), positive predictive value (+LR) (Equation 8), and negative predictive value (-LR) (Equation 9). They are defined as follows:


(3) $$Sen=\frac{TP}{TN+FP}$$

(4) $$Spe=\frac{TN}{TN+FP}$$

(5) $$Acc=\frac{TP+TN}{TP+FP+TN+FN}$$

(6) $$PPV=\frac{TP}{TP+FP}$$

(7) $$NPV=\frac{TN}{TN+FN}$$

(8) $$+LR=\frac{Sen}{1-Spe}$$

(9) $$-LR=\frac{1-Sen}{Spe}$$

where TP is the number of true-positive cases, FP the number of false-positive cases, TN the number of true-negative cases, and FN the number of false-negative cases. The Sen, Spe, Acc, PPV, NPV, +LR and -LR of the network model and each DSC were assessed using Python. The Sen, Spe, Acc, PPV, NPV, +LR, and -LR of the original ultrasonic reports were assessed using MedCalc version 10.4.7.0 (USA).

## 3 Results

### 3.1 Baseline characteristics

During the study period, 5620 potential LNs were detected. Of these, 2323 LNs were excluded because of the presence of other diseases, such as breast cancer, laryngocarcinoma, and tuberculosis. A total of 238 LNs were excluded because of the lack of high-quality images. Finally, 3059 LNs from 2398 patients were included, of which 1535 were benign and 1524 were malignant. There were 928 male patients, of whom 409 and 519 had benign and malignant tumors, respectively. The remaining 2131 patients were female, of whom 1126 and 1005 had benign and malignant tumors, respectively. The proportion of malignancies was higher in male than in female patients (*P<* 0.001). The length of the long axis of the LNs ranged from 3 to 37 (mean, 14.8) mm, and the short axis length ranged from 2 to 25 (mean, 6.9) mm.

LNs spread from neck level I to level VII, and levels III and IV were predominant in both benign and malignant cases (**Table 2**).

**Table 2:** The location of the included lymph nodes.

Location	Malignancy	Benignancy
Level I in the right neck	5	9
level I in the left neck	0	5
level II in the right neck	74	100
level II in the left neck	60	122
Level III in the right neck	285	272
level III in the left neck	263	348
level IV in the right neck	310	184
level IV in the left neck	273	328
level V in the right neck	10	44
level V in the left neck	11	33
level VI in the right neck	102	35
level VI in the left neck	105	49
level VII in the neck	26	6

### 3.2 Segmentation results

Of the 3059 patients, 2512 were randomly selected for the training and test sets, and the remaining 547 LNs were divided into the validation dataset. In the validation set, 307 patients had benign and 240 had malignant tumors.

**Figure 2:**
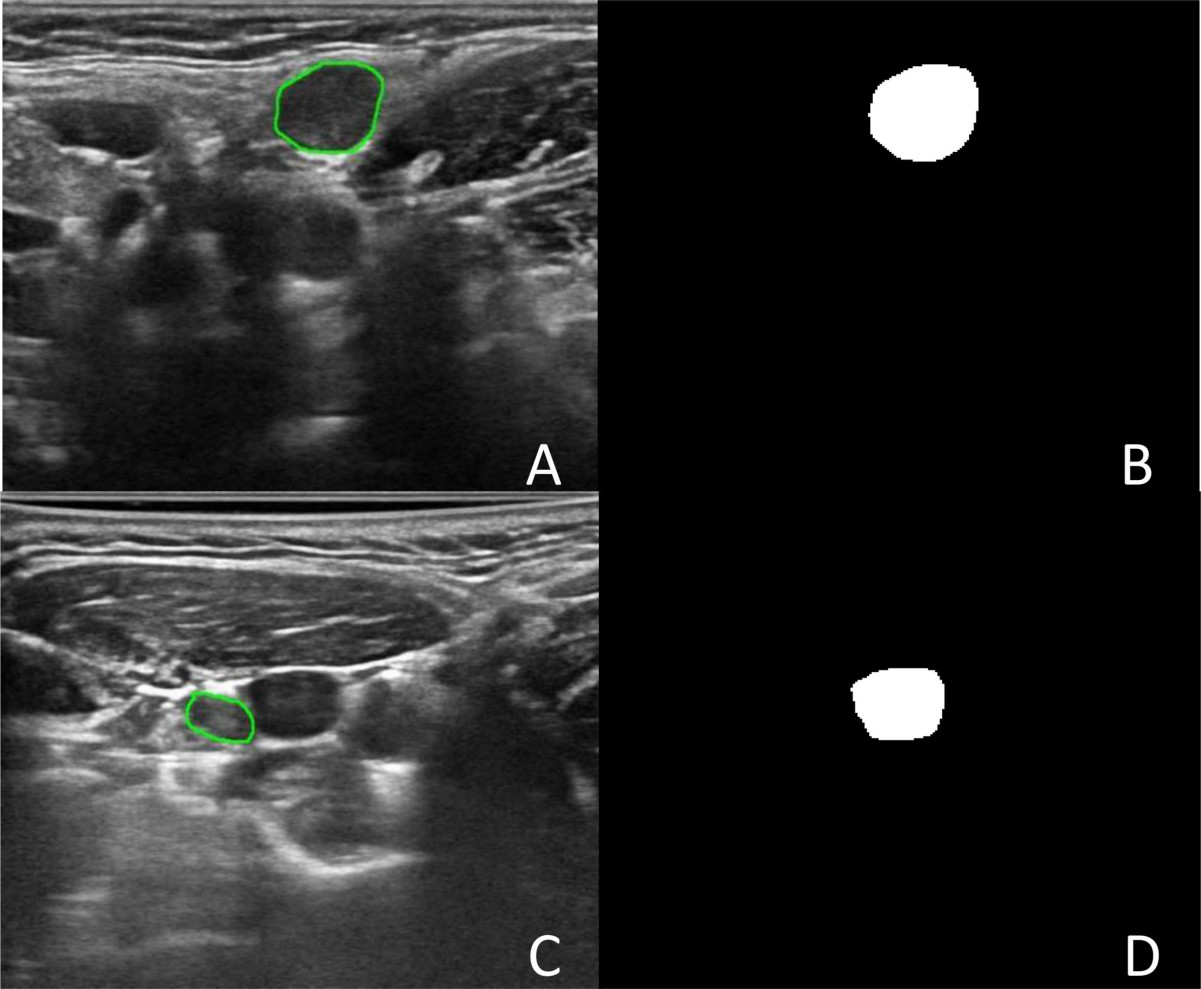
The segmentation of Lymph node. **(A)** Was an image with lymph node in level II in the left neck. **(B)** Was the binary image of automatic segmentation, and the DSC was 0.972. **(C)** Was an image with lymph node in level III in the right neck. **(D)** Was the binary image of automatic segmentation. The jugular vein was incorrectly segmented as lymph node, and the DSC was 0.002.

**Figure 3:**
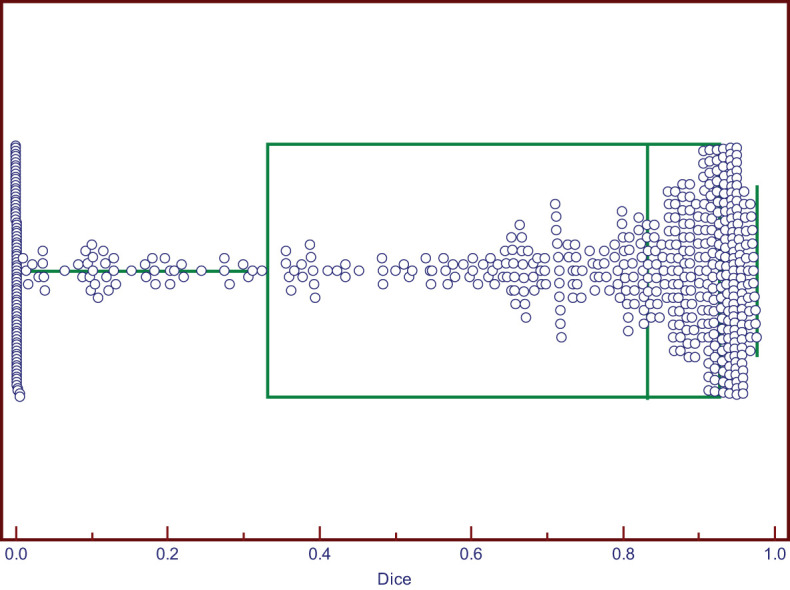
The Box-and-Whisker plot of DSC, the median of DSC was 0.832.

The DSC values ranged from 0 to 0.976. Two cases of good segmentation and poor segmentation are shown in **Figure 2**. A scatter diagram is shown in **Figure 3**. The data did not correspond to a normal distribution. The upper quartile was 0.324, the median was 0.832, and the lower quartile was 0.928.

### 3.3 Classification results

**Figure 4:**
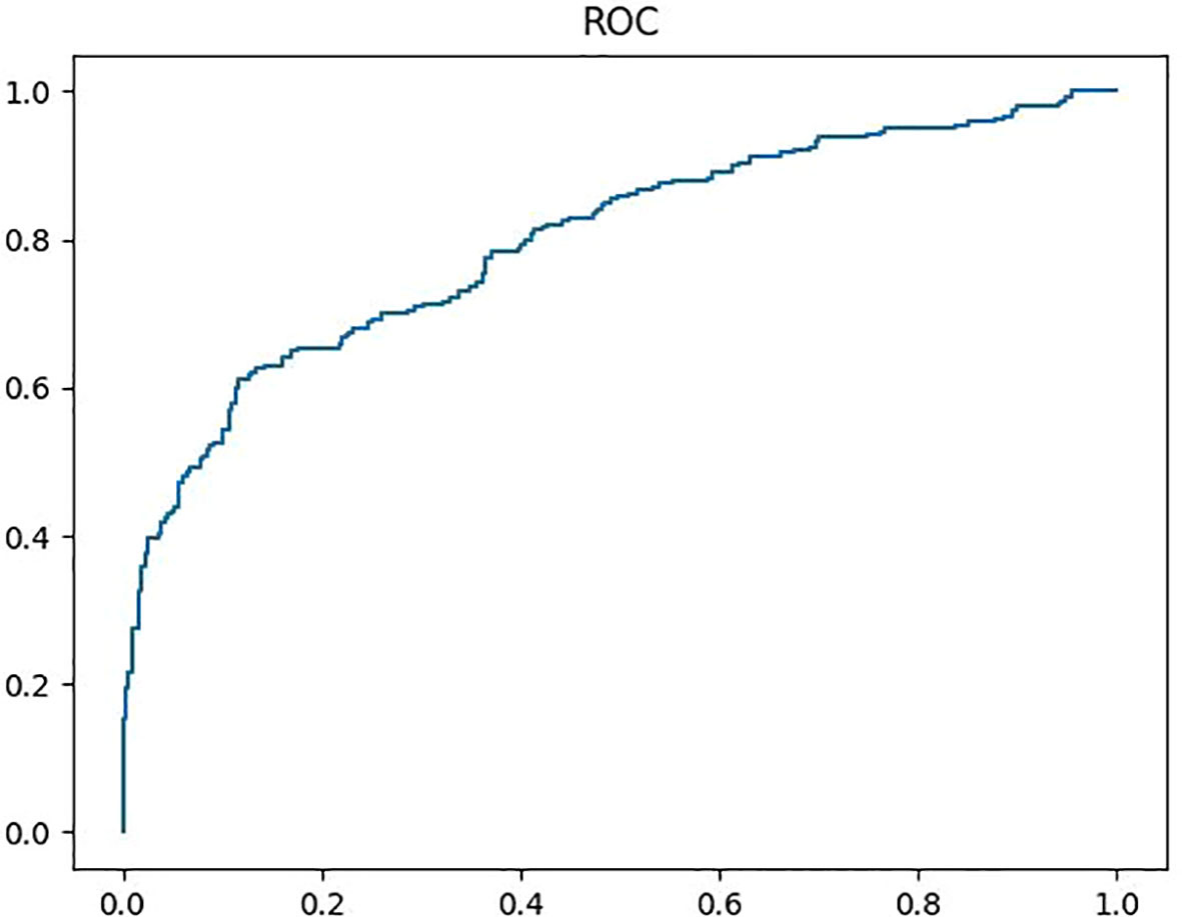
ROC curve of the Y-Net modal, the AUC was 0.797.

The diagnostic efficiencies of the original ultrasonic reports and the Y-Net model are summarized in **Table 3**. The Sen, Spe, Acc, PPV, NPV, +LR, and -LR of the Y-Net model were 57.25%, 87.08%, 72.03%, 81.87%, 66.67%, 4.43, and 0.49, respectively. The AUC (area of curve) of ROC (receiver operating characteristic curve) was 0.797 (**Figure 4**). When only classification were input the Y-Net model, The classification Sen, Spe, Acc, PPV, NPV, +LR, and -LR of 84.78%, 80.23%, 82.45%, 79.35%, 85.61%, 4.29, and 0.19, respectively. For the original ultrasonic reports, the Sen, Spe, Acc, PPV, NPV, +LR, and -LR were 95.14%, 34.3%, 64.66%, 59.02%, 87.71%, 1.45, and 0.14, respectively.

**Table 3:** The diagnostic efficiency of the model and original ultrasonic reports.

	Sen	Spe	Acc	PPV	NPV	+LR	-LR
Y-Net (Classification combined segmentation	57.25%	87.08%	72.03%	81.87%	66.67%	4.43	0.49
Y-Net (Only classification)	84.78%	80.23%	82.45%	79.35%	85.61%	4.29	0.19
The original ultrasonic reports	95.14%	34.30%	64.66%	59.02%	87.71%	1.45	0.14

+ = positive likelihood ratio.- = Negative likelihood ratio.

## 4 Discussion

In recent years, AI-based deep learning systems have become rapidly used and highly reproducible. As such systems are less affected by inter-observer variations, they are well received by sonographers. Currently, some studies have been conducted on thyroid nodule classification using AI (15–17). Commercial thyroid computer-aided diagnosis (CAD) systems have been integrated into US machines for real-time diagnosis, demonstrating an Acc similar to that of an experienced radiologist (18). Deep learning systems for US imaging to detect LN metastases in the neck have rarely been reported.

In this study, we used the Y-Net network for the auxiliary diagnosis of LN metastasis. The Y-Net network was introduced by Mehta, who extended and generalized U-Net by adding a parallel classification branch (19). The U-Net is a well-known segmentation network for biomedical images (14). The U-Net combines multiscale features by splicing the coding and decoding layer features of the same resolution. Therefore, the network can obtain both the global information of the entire image and multiscale image features, which is consistent with the characteristics of medical image analysis. Most previous studies have proven that the U-Net network is useful for the automatic segmentation of medical images. Jin (20) et al. used four different improved U-Net network models to segment ultrasonic images of an oophoroma. The precision performance of the models was > 85%, and the Pearson correlation coefficient was approximately 0.9, which reflected the reliability and robustness of U-Net. The Y-Net network adds a skip connection between the first and last encoding blocks at some spatial resolution in the encoder to improve segmentation. In addition, the anatomical form of the neck is complicated; in one frame, the LN ultrasonic image contains other organs, such as the vessels, nerves, trachea, esophagus, and muscles. Therefore, the segmentation is similar to the scene analysis of image semantic segmentation. In the 2016 ImageNet Scene Analysis Challenge, the pyramid scene analysis network won first place (21), providing an effective global context for pixel-level scene analysis. Therefore, in this study, the spatial and efficient spatial pyramid pooling modules were introduced. The images were pooled into different sizes, the receptive field was increased, and multiscale features were obtained. Together, the local and global clues make the segmentation more accurate.

In this study, we assumed the segmentation region of an experienced human reader to be the gold standard, and the computer results were compared. In an actual test, the segmentation method exhibited good performance. The median Dice coefficient was 0.832. This was because compared with the thyroid focal area, most benign and metastatic LNs in thyroid carcinoma were well-defined, which was convenient for automatic segmentation. Therefore, when the target LNs were correctly identified, the automatic segmentation matched well with manual images. However, although few, some LNs had totally segmented errors, with a 0 value for the DSC. To analyze the reason for poor segmentation, our team compared the output segmented images and manually labeled images. The reasons for this are summarized as follows. First, some LNs were small, and the surrounding tissues of the CLN were complex, which interfered with the detection of LNs. Second, some surrounding tissues, such as muscle bundles and blood vessels, were incorrectly segmented as LNs because of their similar shapes or echogenicities. In some cases, there were several LNs in one ultrasonic frame. Only one LN of the FNA was the target lesion, and manual segmentation was performed. However, more LNs were mistaken for target lesions and automatically segmented in the Y-Net network, which resulted in a reduction in the Dice coefficient. Therefore, in the future, we will gather negative samples to improve the segmentation efficiency.

The Y-Net model has two parallel output branches of segmentation and classification, which can simultaneously segment and classify images. When both segmentation and classification were performed, the Acc was 72.03%, which was better than that of the original ultrasonic report (64.66%). Therefore, this model would be helpful in improving the diagnostic efficiency of sonographers.

In previous studies, more reports relied on manual thyroid nodules segmentation to predict metastasis to the CLNs, and clinical factors such as sex, patient age, and tumor size were associated with metastasis of CLN (22, 23). Only one study has focused on differentiating benign and malignant CLN of TC. Lee developed a CAD system based on annotated images of LNs in TC (24). The CAD system achieved a Sen of 89.0%, a Spe of 77.0%, and an Acc of 83.0%. In contrast to Lee’s study, the US images in our study were original without annotation, and the result was similar, with the 84.78% sensitivity, 80.23% specificity, and 82.45% accuracy. The result was generally consistent with previous investigations. There are some reports on metastatic LNs using other imaging methods (25–28), consistent with our findings, and most of the reported studies achieved similar Acc. The Acc showed better than radiologists diagnosis, which proved that AI systems are useful screening tools to assist radiologists in more accurate analyses.

This study has some limitations. First, this was a single-center study, although, to the best of our knowledge, it analyzed the largest sample. Multicenter studies are also required to improve the robustness and accuracy of model classification. Second, the included patients were confirmed pathologically and LNs that were followed up were excluded. Therefore, there may have been a selection bias.

## 5 Conclusions

This study attempted to use the Y-Net network to realize the automatic segmentation and classification of CLNs. The experimental results suggest that the deep learning model can be used as a support in clinical practice to improve the diagnostic accuracy of sonographers.

## Data availability statement

The original contributions presented in the study are included in the article/supplementary material. Further inquiries can be directed to the corresponding authors.

## Ethics statement

This study was approved by the Ethics Committee of West China hospital in Sichuan University (No.1341). Written informed consent was not required since this was a retrospective study. All the data set were fully anonymized.

## Author contributions

Conception and design of the study: HZ, and YP. Ultrasound data acquisition: HZ, JL, LS. Clinical and pathological data collection: HZ, BM. Analysis and interpretation of data: HZ, HY. Drafting the manuscript: HZ. Revising and final approval of the version to be published: HZ, YP, BM. All authors contributed to the article and approved the submitted version.

## References

[1] Jan S Laba T-L Essue BM Gheorghe A Muhunthan J Engelgau M. Action to address the household economic burden of non-communicable diseases. Lancet (2018) 391(10134):2047–58. doi: 10.1016/S0140-6736(18)30323-4 29627161

[2] Zheng R Zhang S Zeng H Wang S Sun K Chen Ru. Cancer incidence and mortality in China, 2016. J Natl Cancer center (2022) 2(1):1–9. doi: 10.1016/j.jncc.2022.02.002 PMC11256658 39035212

[3] Chung SR Baek JH Rho YH Choi YJ Sung T-Y Song DE. Sonographic diagnosis of cervical lymoh node metastasis in patients with thyroid cancer and comparison of European and Korean guidelines for stratifying the risk of Malignant lymph node. Korean J Radiol (2022) 23(11):1102–11. doi: 10.3348/kjr.2022.0358 PMC9614289 36126955

[4] Chung SR Baek JH Choi YJ Sung T-Y Song DE Kim TY. Risk factors for metastasis in indeterminate lymph nodes in preoperative patients with thytoid cancer. Eur Radiol (2022) 32(6):3863–8. doi: 10.1007/s00330-021-08478-5 34989848

[5] Kim E Park JS Son K-R Kim JH Jeon SJ Na DG. Preoperative diagnosis of cervical metastatic lymph nodes in papillary thyroid carcinoma: comparison of ultrasound, computed tomography , and combined ultrasound with computed tomography. Thyroid (2008) 18(4):411–8. doi: 10.1089/thy.2007.0269 18358074

[6] Ryu KH Lee KH Ryu J Baek HJ Kim SJ Jung HK. Cervical lymph node imaging reporting and data system for ultrasound of cervical lymphadenopathy: A pilot study. AJR (2016) 206:1286–91. doi: 10.2214/AJR.15.15381 27070179

[7] Lee HJ Yoon DY Seo YL Kim JH Baek S Kim KJ. Intraobserver and interobserver variability in ultrasound measurements of thyroid nodules. J Ultrasound Med (2018) 37:173–8. doi: 10.1002/jum.14316 28736947

[8] Teng-Fei Yu Wen He Cong-Gui G Zhao M-C Zhu Q Zhang W. Deep learning applied to two-dimensional color Doppler flow imaging ultrasound images significantly improves diagnostic performance in the classification of breast masses: a multicenter study. Chin Med J (2021) 134(4):415–24. 10.1097/CM9.0000000000001329 PMC7909320 33617184

[9] Gao Y Zeng S Xu X Li H Yao S Song K. Deep learning-enabled pelvic ultrasound images for accurate diagnosis of ovarian cancer in China: a retrospective, multicentre diagnostic study. Lancet Digit Health (2022) 4(3):e179–87. doi: 10.1016/S2589-7500(21)00278-8 35216752

[10] Chang L Zhang Y Zhu J Hu L Wang X Zhang H. An integrated nomogram combining deep learning, clinical characteristics and ultrasound features for predicting central lymph node metastasis in papillary thyroid cancer: a multicenter study. Front Endocrinol (2023) 14:964074. doi: 10.3389/fendo.2023.964074 PMC9990492 36896175

[11] Wang J Dong C Zhang Y Wang L Yuan X He M. A novel approach to quantify calcifications of thyroid nodules in US images based on deep learning: predicting the risk of central lymph node metastasis in papillary thyroid cancer patients. Eur Radiol (2023) 33(12):9347–56. doi: 10.1007/s00330-023-09909-1 37436509

[12] He K Zhang X Ren S Sun J. Deep residual learning for image recognition. In: Proceedings of the IEEE conference on computer vision and pattern recognition (2016), 770–778.

[13] Mehta S Rastegari M Caspi A Shapiro L Hajishirze H. ESPNet: Efficient spatial pyramid of dilated convolutions for semantic segmentation. Cham: Springer (2018). doi: 10.1007/978-3-030-01249-6_34

[14] Ronneberger O Fischer P Brox T. U-Net: Convolutional networks for biomedical image segmentation. Springer international publishing (2015).

[15] Zhang Q Zhang S Pan Y Sun L Li J Qiao Y. Deep learning to diagnose Hashimoto’s thyroiditis from sonographic images. Nat Commun (2022) 13(1):3759. doi: 10.1038/s41467-022-31449-3 35768466 PMC9243092

[16] Zhang B Jin Z Zhang S. A deep-learning model to assist thyroid nodule diagnosis and management. Lance Digit Health (2021) 3(7):e409. doi: 10.1016/S2589-7500(21)00108-4 34120887

[17] Tao Y Yu Y Wu T Xu X Dai Q Kong H. Deep learning for the diagnosis of suspicious thyroid nodules based on multimodal ultrasound images. Front Oncol (2022) 12:1012724. doi: 10.3389/fonc.2022.1012724 36425556 PMC9680169

[18] Choi YJ Baek JH Park HS Shim WH Kim TY Shong YK. A computer-aided diagnosis system using artificial intelligence for the diagnosis and characterization of thyroid nodules on ultrasound: initial clinical assessment. Thyroid (2017) 27:546–52. doi: 10.1089/thy.2016.0372 28071987

[19] Mehta S Mercan E Bartlett J Weave D Elmore JG Shapiro L. Y-Net: joint segmentation and classification for diagnosis of breast biopsy images. Comput Vision Pattern recognition (2018) 552742. doi: 10.1007/978-3-030-00934-2_99

[20] Jin J Zhu H Zhang J Ai Y Jin X. Multiple U-Net based automatic segmentations and radiomics feature stability on ultrasound images for patients with ovarian cancer. Front Oncol (2020) 10:614201. doi: 10.3389/fonc.2020.614201 33680934 PMC7930567

[21] Zhao H Shi J Qi X Wang X Jia J. Pyramid scene parsing network. In: CVPR (2017).

[22] Wang Z Qu L Chen Q Zhou Y Duan H Li B. Deep learning-based multifeature integration robustly predicts central lymph node metastasis in papillary thyroid cancer. BMC Cancer (2023) 23(1):128. doi: 10.1186/s12885-023-10598-8 36750791 PMC9906958

[23] Fu R Yang H Zeng D Yang S Luo P Yang Z. PTC-MAS: a deep learning-based preoperative automatic assessment of lymph node metastasis in primary thyroid cancer. Diagnostics (2023) 13(10):1723. doi: 10.3390/diagnostics13101723 37238205 PMC10217266

[24] Lee JH Baek JH Kim JH Shim WH Chung SR Choi YJ. Deep learning-based computer-aided diagnosis system for localization and diagnosis of metastatic lymph nodes on ultrasound : A pilot study. Thyroid (2018) 28(10):1332–8. doi: 10.1089/thy.2018.0082 30132411

[25] Abbasian Ardakani A Mohammadi A Mirza-Aghazadeh-Attari M Faeghi F Vogl TJ Acharya UR. Diagnosis of metastatic lymph nodes in patients with papillary thyroid cancer: a comparative multi-center study of semantic features and deep learning based models. J Ultrasoind Med (2022) 27:16131. 10.1002/jum.16131 36437513

[26] Lee JH Ha EJ Kim JH Jung YJ Heo S Jang YH. Application of deep learning to the diagnosis of cervical lymph node metastasis from thyroid cancer with CT: external validation and clinical utility for resident training. Eur Radiol (2020) 30(6):3066–72. doi: 10.1007/s00330-019-06652-4 32065285

[27] Lee JH Ha EJ Kim JH Jung YJ Heo S Jang YH. Application of deep learning to the diagnosis of cervical lymph node metastasis from thyroid cancer with CT. Eur Radiol (2019) 29(10):5452–7. doi: 10.1007/s00330-019-06098-8 30877461

[28] Kavitha M Lee C-H KattakkaliSubhashdas K Ahn BC. Deep learning enables automated localization of the metastatic lymph node for thyroid cancer on 131I post-ablation whole-body planar scans. Sci Rep (2020) 8(10):7738. 10.1038/s41598-020-64455-w PMC7211007 32385375

